# Long-term response to genomic selection: effects of estimation method and reference population structure for different genetic architectures

**DOI:** 10.1186/1297-9686-44-3

**Published:** 2012-01-24

**Authors:** John WM Bastiaansen, Albart Coster, Mario PL Calus, Johan AM van Arendonk, Henk Bovenhuis

**Affiliations:** 1 Animal Breeding and Genomics Centre, Wageningen University, P.O. Box 338, 6700 AH, Wageningen, the Netherlands; 2 Animal Breeding and Genomics Centre, Animal Science Group, Lelystad, the Netherlands

## Abstract

**Background:**

Genomic selection has become an important tool in the genetic improvement of animals and plants. The objective of this study was to investigate the impacts of breeding value estimation method, reference population structure, and trait genetic architecture, on long-term response to genomic selection without updating marker effects.

**Methods:**

Three methods were used to estimate genomic breeding values: a BLUP method with relationships estimated from genome-wide markers (GBLUP), a Bayesian method, and a partial least squares regression method (PLSR). A shallow (individuals from one generation) or deep reference population (individuals from five generations) was used with each method. The effects of the different selection approaches were compared under four different genetic architectures for the trait under selection. Selection was based on one of the three genomic breeding values, on pedigree BLUP breeding values, or performed at random. Selection continued for ten generations.

**Results:**

Differences in long-term selection response were small. For a genetic architecture with a very small number of three to four quantitative trait loci (QTL), the Bayesian method achieved a response that was 0.05 to 0.1 genetic standard deviation higher than other methods in generation 10. For genetic architectures with approximately 30 to 300 QTL, PLSR (shallow reference) or GBLUP (deep reference) had an average advantage of 0.2 genetic standard deviation over the Bayesian method in generation 10. GBLUP resulted in 0.6% and 0.9% less inbreeding than PLSR and BM and on average a one third smaller reduction of genetic variance. Responses in early generations were greater with the shallow reference population while long-term response was not affected by reference population structure.

**Conclusions:**

The ranking of estimation methods was different with than without selection. Under selection, applying GBLUP led to lower inbreeding and a smaller reduction of genetic variance while a similar response to selection was achieved. The reference population structure had a limited effect on long-term accuracy and response. Use of a shallow reference population, most closely related to the selection candidates, gave early benefits while in later generations, when marker effects were not updated, the estimation of marker effects based on a deeper reference population did not pay off.

## Background

Genomic breeding values estimated with genetic markers distributed over the whole genome (MEBV) have become important in dairy cattle breeding [1,2], and efforts are undertaken to implement this technology in other animal species [3,4] as well as in plants [5,6]. The expected advantages of selection based on MEBV over traditional selection methods, where the estimation of breeding values is based solely on phenotypes and pedigree information, include an increased accuracy of MEBV compared to traditionally estimated breeding values, in combination with a reduced generation interval and a lower rate of inbreeding, e.g. due to the ability to distinguish between sibs [7-11].

Calculation of MEBV requires a population with information on genetic markers and phenotypes, called the *reference *population. Information on the relation between markers and phenotypic information in the reference population is used to calculate MEBV of individuals with only marker information, called the *evaluation *population. Factors affecting the accuracy of MEBV include the heritability of the trait, the size of the reference population, the method used to estimate allelic effects of the markers, linkage disequilibrium (LD) between markers and quantitative trait loci (QTL), and the distribution of QTL effects, i.e. the genetic architecture of the trait [7,12-16].

The accuracy of estimated breeding values, estimated either with traditional methods such as pedigree BLUP or with the use of markers, decreases when the number of generations separating the reference and the evaluation populations increases [7,17]. Using pedigree BLUP, this decrease is mainly due to the inability of this method to predict the random segregation of genomic segments to the next generation. Using markers, this segregation can be traced and, for the part of the genetic variance that is explained through LD with the markers, the decrease in accuracy per generation is smaller than for the remaining part of the genetic variance that is explained solely by family structure. The accuracy that is due to LD with markers is only affected by the changing patterns of LD between markers and QTL. More persistent accuracies of MEBV are expected when the average distance between markers and QTL decreases, as this leads to lower recombination rates [18]. The structure of the reference population is expected to have an effect on the persistence of accuracies because it affects how well the genetic variance of QTL can be assigned to markers near the QTL as opposed to markers that are more distant. When individuals in the reference population are more related, they will share longer stretches of chromosomes surrounding the QTL, allowing more distant markers to explain QTL variation within the reference population. Because the recombination rates between these more distant markers and the QTL are higher, they will loose their predictive value more quickly compared to markers near the QTL. Selecting animals for the reference population across more generations will reduce the average relationship within the reference population and is expected to lead to more persistent accuracies of MEBV. Moreover, in populations under selection, LD is expected to change more rapidly compared to unselected populations, with the result that accuracies of the MEBV decrease faster under selection [5,11].

A variety of methods for estimating MEBV exist, including Bayesian methods (BM) such as BayesA and BayesB proposed by Meuwissen et al. [7], ridge regression [7,11], BLUP methodology with the use of a realized relationship matrix calculated from the markers (GBLUP) [1,19], principal component regression (PCR) [15], and partial least square regression (PLSR) [15,16].

Methods BM and PLSR deal with the high dimension of the marker data by assigning different variances or weights to individual markers. After one generation, these methods result in higher accuracies when genetic variance is due to a small number of QTL compared to traits with more QTL of small effect [16,20]. Pedigree BLUP and GBLUP estimate covariances between individuals based on pedigree data or marker data, respectively and may be less dependent than BM and PLSR on LD between individual markers and QTL [14].

The performance of estimation methods has been extensively evaluated in simulations. Information on the performance of these methods when the MEBV are being used for selection, however, is very limited. A few studies applying selection on MEBV are the selection on MEBV estimated using GBLUP, in the studies of Muir [11]) and Jannink [5] and in a study by Sonesson and Meuwissen [17]. A systematic comparison between methods to calculate MEBV is lacking concerning their ability to achieve a selection response for more than one generation under a range of genetic architectures (number of QTL and distribution of QTL variance).

The objective of this study was to evaluate the impact of choices that can be made, in terms of evaluation methods and between reference population structures, on the long-term selection response. The evaluation was done across a range of genetic architectures to avoid conclusions that may hold only under specific circumstances. The reference population structure was evaluated because a reference population made up of multiple generations was expected to increase the long-term accuracy of MEBV compared to a reference population made up of a single generation [11,17,21]. Comparisons of methods and reference structures were based on genetic progress, accuracy of MEBV, inbreeding rate and reduction of genetic variance. Finally, accuracies of MEBV under directional selection were compared to accuracies with random selection.

## Methods

### Simulation of data and estimation methods

The simulations were performed using the R-package HaploSim [22], which is available from the R repository CRAN at http://cran.r-project.org/package=HaploSim. We refer to Coster et al. [16] for a detailed description of the simulations to create the starting populations. Briefly, the simulated genomes consisted of four 1 Morgan chromosomes. The genome contained 40 000 equally distributed loci where mutations were allowed, most of the 40 000 loci were monomorphic at any time. Random mating was simulated from generation -5005 to generation -1 to generate LD between loci and to reach mutation-drift equilibrium. The number of recombinations on each chromosome per meiosis event was drawn from a Poisson distribution, and the mutation rate of the 40 000 loci was set at 10^-5 ^per meiosis. The mutation rate was set to 0 after generation -1, to avoid the introduction of a large number of markers with very low minor allele frequency (MAF). All loci that were polymorphic in generation -1 were used as markers. Each individual in generations -5005 to -2 contributed two gametes to the next generation, which were randomly combined to form individuals. Consequently, a constant population size of 100 individuals with an effective population size of 199 was maintained throughout these generations of random mating. In generation -1, each individual contributed ten gametes to the next generation, with the objective to increase the population size to 500 individuals. The individuals of this generation were formed as pairs of random gametes from distinct parents to avoid selfing.

Thirty replicates of this population were simulated and stored. From the data of each replicate, all four genetic architectures were created. Each of the five estimation methods was then applied in combination with one or two selection approaches to each population. In this way, identical base populations were used in a variety of simulation and selection scenarios. The four genetic architectures, five estimation methods and two selection approaches are explained below.

Four traits with different genetic architectures were created in each simulated population by combining a *low *or *high *number of QTL, with one of two distributions of QTL variance, *unequal *and *equal *QTL variance (Table 1).

**Table 1:** Genomic selection scenarios

Scenario	Number of QTL	QTL variance	reference population
1	Low	Unequal	1 × 500
2			5 × 100
3		Equal	1 × 500
4			5 × 100
5	High	Unequal	1 × 500
6			5 × 100
7		Equal	1 × 500
8			5 × 100

Combinations of genetic architecture (number of QTL and distribution of QTL variance) and structure of reference population

The high number of QTL was simulated by selecting 50% of the markers with a MAF above 0.10 in generation -4 as QTL. The low number of QTL was simulated by retaining every 10^*th *^QTL from the high QTL density and removing the remaining 90% from the data. QTL density and number of QTL are interchangeable measures because the length of the genome is fixed and the distribution and number of polymorphic loci are the same in all scenarios within a replicate.

The variance of all QTL was set to 1 in the equal distribution case and the allelic effect of a QTL was calculated as $a=\sqrt{\frac{1}{2pq}}$ where p and q are the frequencies of the two QTL alleles. In the unequal distribution case, the allelic effect of every 10^*th *^QTL was multiplied by 9 to make its variance 81 times the variance of the other QTL. This resulted in the unequal distribution, where 10% of the QTL accounted for 90% of the total genetic variance.

All polymorphic loci that remained after selecting the QTL for the high QTL density were used as biallelic markers in all scenarios. Within a replicate, this resulted in an identical set of markers for each genetic architecture.

The true breeding value of an individual was calculated as the sum of the effects of the QTL alleles it received. The additive genetic variance, $\sigma_{\text{a}}^{2}$, was calculated as the variance of the breeding values of the individuals in generation -4. Random normal deviates from a $\text{N}\left(0,\sigma_{\text{e}}^{2}\right)$ distribution were added to the breeding values to simulate phenotypes with a heritability of 0.25.

The reference population always consisted of 500 individuals with genotypes and phenotypes but could have one of two structures. The reference population was either *shallow*, consisting of all 500 individuals from generation 0 (1 × 500), or the reference population was *deep*, consisting of 100 individuals from each of generations -4 to 0 (5 × 100). The deep reference population was an attempt to reduce the average relationship between reference animals compared to the shallow reference population. In generations following those of the reference population, no additional phenotypes were recorded for methods BM, PLSR and GBLUP, and therefore the marker effects were not updated after the initial analysis of the reference population.

Breeding values were estimated for all individuals from generation -4 onwards using five different methods. The first two methods were a bayesian model (BM) and partial least square regression (PLSR). BM and PLSR methods were similar in that they estimated allelic effects for each individual marker using the phenotypes and markers in the reference population. These estimated allelic effects were subsequently used to calculate MEBV as follows:

(1) $$\mathrm{MEBV}=X\hat{a}$$

where **MEBV **was the vector of breeding values estimated with the marker genotypes, **X **was an incidence matrix that related allele counts to individuals, and **â **was the vector of allelic effects of the markers, estimated either with method BM or with PLSR.

The next two methods applied the BLUP methodology. Genomic BLUP (GBLUP) used a relationship matrix, **G**, estimated from marker data and pedigree BLUP (BLUP) estimated the relationship matrix, **A**, from pedigree records. Both GBLUP and BLUP used **G **or **A **as a covariance matrix among relatives in an animal model:

(2) $$\begin{array}{c} \mathrm{EBV}=Z\hat{u}\\ \;\;\hat{u}\sim N\left(0,G\sigma_{a}^{2}\right)\;\text{or}\\ \;\;\hat{u}\sim N\left(0,A\sigma_{a}^{2}\right) \end{array}$$

where **EBV **was a vector of estimated breeding values (EBV for estimates from pedigree BLUP and MEBV for estimates from GBLUP which used the marker data), **Z **was an incidence matrix relating each individual to its breeding value in vector **û**.

In the last method, RANDOM, random numbers were assigned to selection candidates as estimated breeding values. This method was included as a baseline in which changes in LD are only affected by drift and recombination. The RANDOM method made it possible to compare changes in accuracies of MEBV over generations in situations with and without selection acting on LD and allele frequencies.

#### Bayesian method

The Bayesian method (BM) was used as implemented by Verbyla et al. [23]. In this model, the allelic effects of the markers were considered independent random normal variables. The allelic effects of markers were considered to be from a mixture distribution. Effects were sampled from a wide $N\left(0,\sigma_{1}^{2}\right)$ distribution or a more narrow $N\left(0,\sigma_{1}^{2}/100\right)$ distribution. The prior for the probability of marker effect being sampled from the wide distribution was the ratio of the true number of QTL over the number of markers. The true number of QTL was counted in the generations that contributed to the reference population, generations -4 to 0. The prior for the QTL variance $\sigma_{1}^{2}$ was set to the genetic variance resulting in generations -4 to 0, divided by the true number of QTL. The priors were set separately for each scenario and each replicate. The BM method used Gibbs sampling to numerically integrate over the posterior distribution of the model. The Gibbs sampler was run for 10 000 iterations and the first 1000 iterations were discarded as burn-in. Estimates of allelic effects of the markers were calculated as the mean of the posterior distributions.

#### Partial least square regression

Partial Least Square Regression (PLSR) reduces the dimensions of the regression model by building orthogonal linear combinations of markers, or components, which have a maximal correlation with the trait [24]. The trait was subsequently regressed on these components. Cross-validation was used on the data in the reference population to find the number of components that minimized the prediction error. We used the plsr function in the package pls [25] of R [26] to fit and cross-validate the models. The algorithm to fit and cross-validate the PLSR models was modified according to Coster et al. [16] to reduce the computation time.

#### GBLUP method

GBLUP was performed by solving the mixed model equations of an animal model using a relationship matrix estimated from the marker data as the covariance matrix among relatives, following Van Raden [27]. The relationship matrix **G **was calculated as:

(3) $$G=\mathrm{MD}M^{t},$$

where matrix **M **was the genotype matrix, with -1 for one of the homozygous genotypes, 0 for a heterozygous genotype and 1 for the alternative homozygous genotype. Matrix **D **was a diagonal matrix with the reciprocal of the expected variance of each marker on the diagonal elements $\left(\frac{1}{2\mathrm{pq}}\right)$ where p and q were the frequencies of the two QTL alleles. We used the gblup function in the pedigree package [28] of R [26] to calculate these MEBV, using the simulated heritability of 0.25.

#### Pedigree BLUP method

The simulated phenotypes of all 900 individuals in generations -4 to 0 were used to estimate breeding values using pedigree BLUP. This represents 400 additional phenotypes compared to the 500 used by all three genomic estimation methods. The inverted genetic relationship matrix **A**^-1 ^was calculated from the pedigree data with generation -4 as the unrelated base population. The matrix **A**^-1 ^was calculated using function makeAinv of the R-package pedigree. The pedigree BLUP approach only used phenotypes of the 900 individuals in generations -4 to 0. For the subsequent generations, only pedigree information was used to estimate breeding values. We used the blup function of the pedigree package in R [28] to calculate the EBV, with the simulated level of heritability of 0.25.

### Selection

Selection started in generation 0, the last generation of the reference data, and was continued for ten generations. In each generation, 100 individuals (50 males and 50 females) were selected from the 500 candidates. Selected individuals were mated at random and each pair produced ten offspring, making the next generation consist of 50 fullsib families of size ten.

The three methods to calculate MEBV (BM, PLSR, GBLUP) were applied to each of the two reference population structures to form six genomic selection approaches. Each genomic selection approach was applied to each of the four genetic architectures (Table 1). Selection on pedigree BLUP EBV and RANDOM selection were also applied to each of the four genetic architectures.

In the RANDOM scenarios, selection was performed by randomly sampling males and females to produce the next generation. Breeding values of random selection generations were estimated with each of the three genomic estimation methods BM, PLSR and GBLUP. The random selection scenario was included to assess the impact of selection on accuracies of MEBV. Accuracies of MEBV from selection scenarios were compared to accuracies of MEBV in the RANDOM scenarios where there was no selection that could cause changes in the LD between markers and QTL, changes in the frequencies of QTL alleles, or reduction of $\sigma_{\text{G}}^{2}$. This resulted in 32 unique scenarios of genetic architecture by selection approach. The results for each scenario were obtained from 30 replicates.

### Comparing selection approaches

The evaluation of simulation scenarios was based on genetic improvement, incurred inbreeding, accuracy of the (M)EBV and loss of genetic variance over ten generations of selection. Genetic improvement was calculated as the cumulative increase of the average breeding value ($\bar{\text{G}}$) in each generation. The average breeding value in each generation was standardized and presented as a percentage of the genetic standard deviation in generation 0.

The inbreeding coefficient was calculated for each individual in the pedigree using the function calcInbreeding from the R-package pedigree [28]. The average increase in inbreeding was calculated for each generation, using generation 0 as the base population. The accuracy of the (M)EBV was calculated as the correlation of these (M)EBV with the simulated breeding values of the individual from each generation. The genetic variance was calculated in each generation as the variance of the simulated breeding values and presented as a percentage of the genetic variance in generation 0 or as the percentage reduction in genetic variance from generation 0.

## Results

### Characteristics of the simulated populations

For each replicate, all 36 scenarios started with the same number of markers in generation 0, which was on average 1429 across the 30 replicates (Table 2). The average minor allele frequency (MAF) of markers was 0.09, reflecting a U-shaped distribution of allele frequencies. Average LD between adjacent markers, measured as *r*^2^, was 0.05 (Table 2). This low *r*^2 ^value was due to the high number of low frequency alleles, which resulted from recent mutations. The average *r*^2 ^between markers with MAF above 0.1, was 0.15, which was in line with expectations based on Sved [18].

**Table 2:** Simulated marker and QTL data

QTL	SNP			QTL			
			
	number	MAF	**r**^**2**^	number	MAF	**r**^**2**^	**R**^**2**^
Low	1429.4 (2.6)	0.09 (0.00)	0.05 (0.01)	34.4 (0.1)	0.27 (0.02)	0.01 (0.00)	0.46 (0.05)
High	1429.2 (2.6)	0.09 (0.00)	0.05 (0.01)	339.9 (1.2)	0.27 (0.01)	0.15 (0.01)	0.47 (0.02)

Average (standard error) of the number, minor allele frequency (MAF), and linkage disequilibrium (*r*^2^) with flanking markers, of markers and QTL and average maximum linkage disequilibrium between a marker and each QTL (*R*^2^); simulated number of QTL was low or high; summary of 30 replicated simulations

The average number of QTL was 34 for the low QTL density and 340 for the high QTL density architecture (Table 2). The average LD between QTL was 0.01 for the low QTL density and 0.15 for the high QTL density architecture (Table 2). The number of QTL that accounted for 90% of the genetic variance ranged from only 3 for the low-unequal architecture to 306 for the high-equal architecture. Linkage disequilibrium between markers and QTL (*R*^2^) was defined as the average *r*^2 ^between each QTL and the marker in highest LD with that QTL. The *R*^2 ^was 0.46 for the low QTL and 0.47 for the high QTL density architecture, reflecting the fact that the marker density was the same in both scenarios (Table 2).

### Response to selection

The increases in average genetic value $\bar{\text{G}}$ and in the average inbreeding $\bar{\text{F}}$ were measured over ten generations of selection. The reductions in the accuracy of MEBV and of $\sigma_{G}^{2}$ during selection were also measured because on the one hand they are affected by past selection and on the other hand they affect the genetic progress that can be obtained with future selection (i.e. ΔG = *i *· *ρ *· *σ*_G_).

Genetic architecture had a strong impact on the maximum increase in $\bar{\text{G}}$ that was reached after ten generations of selection. The maximum increase in $\bar{\text{G}}$ was 321% for the low-unequal architecture and between 372 and 384% for the other three architectures (Table 3). The pattern of much lower levels of $\bar{\text{G}}$ with the low-unequal architecture, compared to the other three genetic architectures, was the same for all estimation methods. The low-unequal architecture showed a fast reduction of genetic variance, indicating that the few QTL were quickly moved towards small minor allele frequencies. The order of the other three genetic architectures for final level of $\bar{\text{G}}$ was not consistent across estimation methods, but differences between these three genetic architectures were generally small. The response to the first generation of selection was similar for the three genomic evaluation methods when compared within a specific genetic architecture (Table 3). Increases of $\bar{\text{G}}$ declined over generations for all selection approaches. For pedigree BLUP, $\bar{\text{G}}$ reached a plateau after about two generations of selection.

**Table 3:** Response to genomic selection

Generation		Unequal		Equal	
			
	Model	Low	High	Low	High
**1**					
	BM 1	93.1 (3.6)	88.1 (1.5)	79.7 (2.0)	86.7 (1.9)
	BM 5	85.4 (4.0)	74.8 (2.2)	66.9 (2.2)	74.4 (2.3)
	PLSR 1	86.5 (2.4)	89.6 (1.9)	86.3 (1.8)	90.0 (1.8)
	PLSR 5	78.5 (2.3)	80.2 (2.3)	76.2 (2.1)	77.0 (3.2)
	GBLUP 1	91.2 (2.3)	93.9 (1.5)	89.0 (1.7)	91.0 (1.3)
	GBLUP 5	75.9 (2.2)	79.4 (2.0)	78.2 (1.6)	77.3 (1.7)
	BLUP	85.0 (1.6)	86.1 (1.1)	85.5 (1.3)	86.0 (1.4)
	RANDOM	-0.3 (1.7)	-1.9 (2.0)	-0.8 (1.4)	0.1 (1.8)

**10**					
	BM 1	312.6 (19.2)	354.3 (16.3)	346.8 (12.6)	366.7 (12.1)
	BM 5	317.7 (17.6)	333.1 (13.8)	343.9 (14.1)	326.3 (14.3)
	PLSR 1	305.0 (17.4)	384.0 (14.4)	379.8 (15.1)	372.4 (11.5)
	PLSR 5	306.1 (15.7)	348.6 (14.4)	364.7 (13.0)	327.5 (19.4)
	GBLUP 1	321.5 (18.2)	365.2 (12.1)	361.5 (13.1)	366.0 (9.6)
	GBLUP 5	298.4 (15.9)	369.2 (11.4)	372.4 (12.4)	367.5 (9.9)
	BLUP	129.9 (11.0)	131.2 (6.5)	136.1 (10.9)	132.9 (12.1)
	RANDOM	-2.1 (6.0)	-9.2 (6.5)	4.4 (6.0)	4.2 (5.0)

Cumulative response (standard deviation), after one and ten generations of selection (as a percentage of the genetic standard deviation in the reference population) in genetic architectures with a low number of QTL of unequal variance (column 3), a high number of QTL of unequal variance (column 4), a low number of QTL of equal variance (column 5) and a high number of QTL of equal variance (column 6); selection was on breeding values estimated with a Bayesian method (BM), partial least square regression (PLSR), genomic BLUP (GBLUP) or pedigree BLUP (BLUP), or selection was at random (RANDOM); numbers 1 and 5 behind estimation methods indicate the number of generations used in the training population

The pattern of results differed between the low-unequal and the other three genetic architectures. In the low-unequal architecture, the BM method was expected to do well because it gives specific emphasis to big QTL. BM was indeed the best genomic selection approach, on average, across reference population structures, both in generation 1 and after ten generations. The three other genetic architectures showed a consistent but different pattern from the low-unequal architecture, with GBLUP performing best in generation 1, while PLSR performed best in generation 10 for approaches that used a shallow reference population, and GBLUP performed best in generation 10 for approaches that used a deep reference population (Table 3).

In generation 1, selection on MEBV from a shallow reference population always resulted in a greater response in $\bar{\text{G}}$ compared to selection on MEBV from a deep reference population (Table 3). Only in a few scenarios did we observe the expected superiority in level of $\bar{\text{G}}$ from a deep reference population after long-term selection, but the differences in levels of $\bar{\text{G}}$ between the deep and shallow reference populations were small for all scenarios.

### Inbreeding

The accumulation of $\bar{\text{F}}$ was always below 1% per generation, except for selection on pedigree BLUP EBV for which the increase in $\bar{\text{F}}$ was 1.7% per generation. No differences in accumulation of $\bar{\text{F}}$ were seen between the different genetic architectures (Table 4). Besides the high inbreeding with the pedigree BLUP selection method, the highest levels of $\bar{\text{F}}$ were incurred with the PLSR and BM selection approaches for all genetic architectures, with $\bar{\text{F}}$ after ten generation ranging from 7.0% to 7.7% for PLSR and from 6.9% to 7.6% for BM. Random selection only incurred a $\bar{\text{F}}$ of 4.7% to 4.9% after ten generations. GBLUP incurred only 1.4% to 1.7% more inbreeding after ten generations than random selection and incurred 0.6% to 0.9%, or roughly one tenth, less inbreeding than PLSR and BM (Table 4). No effect on the accumulation of inbreeding was observed from differences in reference population structure or genetic architecture.

**Table 4:** Inbreeding in genomic selection

Generation		Unequal		Equal	
			
	Model	Low	High	Low	High
**1**					
	BM 1	0.8 (<0.1)	1.0 (<0.1)	0.8 (<0.1)	1.0 (<0.1)
	BM 5	0.9 (<0.1)	0.9 (<0.1)	0.9 (<0.1)	0.8 (<0.1)
	PLSR 1	1.0 (<0.1)	1.0 (<0.1)	1.0 (<0.1)	0.9 (<0.1)
	PLSR 5	0.9 (<0.1)	0.9 (<0.1)	0.9 (<0.1)	0.9 (<0.1)
	GBLUP 1	0.7 (<0.1)	0.8 (<0.1)	0.9 (<0.1)	0.8 (<0.1)
	GBLUP 5	0.9 (<0.1)	0.8 (<0.1)	0.8 (<0.1)	0.9 (<0.1)
	BLUP	0.8 (<0.1)	0.7 (<0.1)	0.8 (<0.1)	0.9 (<0.1)
	RANDOM	0.4 (<0.1)	0.5 (<0.1)	0.4 (<0.1)	0.5 (<0.1)

**10**					
	BM 1	7.1 (<0.1)	7.6 (0.1)	7.1 (0.1)	7.2 (0.1)
	BM 5	7.2 (0.1)	6.9 (0.1)	7.0 (0.1)	7.0 (0.1)
	PLSR 1	7.4 (0.1)	7.7 (0.2)	7.4 (0.2)	7.6 (0.2)
	PLSR 5	7.0 (0.1)	7.3 (0.1)	7.3 (0.1)	7.2 (0.2)
	GBLUP 1	6.2 (<0.1)	6.5 (<0.1)	6.5 (<0.1)	6.4 (<0.1)
	GBLUP 5	6.3 (<0.1)	6.6 (<0.1)	6.4 (<0.1)	6.6 (<0.1)
	BLUP	16.6 (0.3)	17.2 (0.3)	17.1 (0.3)	16.9 (0.3)
	RANDOM	4.9 (<0.1)	4.8 (<0.1)	4.8 (<0.1)	4.7 (<0.1)

Cumulative change (standard deviation) in level of inbreeding, after one and ten generations of selection (as a percentage) in genetic architectures with a low number of QTL of unequal variance (column 3), a high number of QTL of unequal variance (column 4), a low number of QTL of equal variance (column 5) and a high number of QTL of equal variance (column 6); selection was on breeding values estimated with a Bayesian method (BM), partial least square regression (PLSR), genomic BLUP (GBLUP) or pedigree BLUP (BLUP), or selection was at random (RANDOM); numbers 1 and 5 behind estimation methods indicate the number of generations used in the training population

### Accuracy

Accuracies obtained within the reference population were similar for all the genomic estimation methods, with an average of 0.63 ± 0.03. For all scenarios, the accuracies dropped steeply in the first generations of selection, after which the decline became more or less linear. After ten generations of selection with the low-unequal genetic architecture, all genomic selection approaches showed an accuracy between 0.07 and 0.10. For the three other genetic architectures, the accuracy after ten generations was only slightly higher, with values between 0.12 and 0.16.

The shallow reference population structure resulted in higher accuracies (0.63 ± 0.03) in the first generation of selection candidates compared to the deep reference population (0.55 ± 0.03). In the shallow reference structure, all selection candidates were included in the reference population with own phenotypes while in the deep reference structure, only 20% of the selection candidates were included in the reference population with own phenotypes. In generation 10, however, accuracies were no longer different between the two structures for a given genetic architecture and estimation method (Table 5).

**Table 5:** Accuracy of genomic selection

Generation		Unequal		Equal	
			
	Model	Low	High	Low	High
**1**					
	BM 1	0.47 (0.04)	0.37 (0.01)	0.31 (0.02)	0.35 (0.02)
	BM 5	0.48 (0.04)	0.32 (0.01)	0.31 (0.01)	0.33 (0.01)
	PLSR 1	0.40 (0.02)	0.38 (0.01)	0.39 (0.01)	0.37 (0.02)
	PLSR 5	0.37 (0.02)	0.35 (0.02)	0.35 (0.01)	0.35 (0.02)
	GBLUP 1	0.38 (0.01)	0.37 (0.01)	0.35 (0.01)	0.36 (0.01)
	GBLUP 5	0.35 (0.01)	0.35 (0.01)	0.32 (0.01)	0.35 (0.01)
	BLUP	0.23 (0.02)	0.24 (0.02)	0.22 (0.01)	0.22 (0.01)

**10**					
	BM 1	0.08 (0.01)	0.12 (0.01)	0.15 (0.01)	0.14 (0.01)
	BM 5	0.05 (0.02)	0.13 (0.02)	0.11 (0.01)	0.13 (0.01)
	PLSR 1	0.08 (0.02)	0.11 (0.02)	0.15 (0.02)	0.15 (0.01)
	PLSR 5	0.09 (0.01)	0.11 (0.02)	0.13 (0.02)	0.12 (<0.01)
	GBLUP 1	0.08 (0.01)	0.14 (0.02)	0.14 (0.01)	0.14 (0.01)
	GBLUP 5	0.08 (0.01)	0.13 (0.01)	0.15 (0.02)	0.15 (0.01)
	BLUP	0.01 (0.02)	0.01 (0.03)	0.02 (0.03)	0.00 (0.03)

Accuracy (standard deviation), after one and ten generations of selection in genetic architectures with a low number of QTL of unequal variance (column 3), a high number of QTL of unequal variance (column 4), a low number of QTL of equal variance (column 5) and a high number of QTL of equal variance (column 6); selection was on breeding values estimated with a Bayesian method (BM), partial least square regression (PLSR), genomic BLUP (GBLUP) or pedigree BLUP (BLUP); numbers 1 and 5 behind estimation methods indicate the number of generations used in the training population

**Figure 1:**
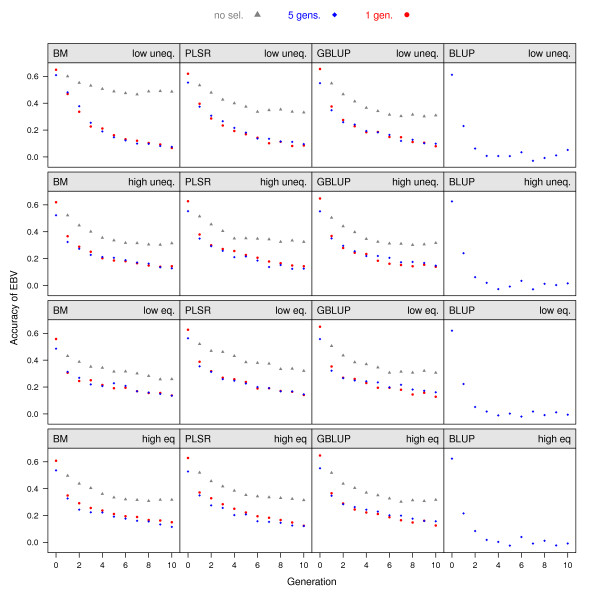
**Accuracy of estimated breeding values**. Accuracy of MEBV in generations 0 to 10 averaged over 30 replicates; panels show results from genetic architectures with a low number of QTL of unequal variance (row 1), a low number of QTL of equal variance (row 2), a high number of QTL of unequal variance (row 3) and a high number of QTL of equal variance (row 4); estimation methods are BM (column 1), PLSR (column 2), GBLUP (column 3) and pedigree BLUP (column 4); levels of accuracy are shown for selection with training on phenotypes from one generation (shallow reference population, red circles) or from five generations (deep reference population, blue diamonds); accuracies of MEBV under RANDOM selection are shown as gray triangles; symbols for some scenarios may be hidden if values overlap.

MEBV were also estimated in each generation of the RANDOM selection scenarios based on training in the shallow reference population. Accuracies in the RANDOM selection scenarios were well above accuracies from the same model when directional selection was applied (Figure 1). The largest difference was seen in the low-unequal genetic architecture where accuracy decreased quickly with the application of selection, primarily due to reduction of genetic variance. In the other three genetic architectures, differences were smaller, but the accuracies for the RANDOM selection scenarios were still 18% higher, on average. In the low-unequal architecture, accuracy was higher after ten generations of RANDOM selection with BM compared to the two other genomic evaluation methods. In this architecture with few QTL, BM could identify markers close to the QTL with a good predictive ability for several generations because recombinations between these markers and the QTL were rare, due to the short distance between them.

### Genetic variance

Similar to the results for accuracy, a much bigger reduction in genetic variance was observed for the low-unequal architecture compared to the three other genetic architectures in all selection approaches. After the first generation of selection, an important reduction was seen in genetic variance for all selection methods (Table 6). After the initial drop of genetic variance in the first generation of selection, a small rebound in genetic variance was seen in some scenarios before variance started to decrease again. This rebound could be partially attributed to the reduced accuracy of selection in later generations, as it was not observed with BM in the low-unequal scenario, where accuracies in generation 1 were substantially higher. Genetic variance can be increased by favorable QTL alleles moving to more intermediate frequencies. In scenarios with lower accuracies, the balance of increasing genetic variance from changing allele frequencies and decreasing variance from selection resulted in a small increase of variance. Genetic variance steadily decreased over the next generations of selection, except in the pedigree BLUP selection method, which became rather ineffective after a few generations, therefore limiting the loss of genetic variance, even though the inbreeding rate was high for this approach.

**Table 6:** Genetic variance and genomic selection

**Gen**.		Unequal		Equal	
			
	Model	Low	High	Low	High
**1**					
	BM 1	-11.4 (5.1)	-14.5 (2.0)	-11.9 (2.1)	-15.0 (1.8)
	BM 5	-7.2 (4.8)	-6.8 (2.2)	-9.5 (2.0)	-8.7 (2.3)
	PLSR 1	-13.6 (4.0)	-14.8 (1.9)	-16.3 (1.9)	-15.6 (2.2)
	PLSR 5	-12.2 (3.9)	-9.6 (2.3)	-11.9 (2.1)	-8.0 (2.3)
	GBLUP 1	-14.6 (4.3)	-14.2 (1.7)	-17.0 (1.5)	-14.9 (2.2)
	GBLUP 5	-10.5 (4.1)	-9.7 (2.5)	-14.8 (2.0)	-11.6 (1.5)
	BLUP	-9.3 (4.4)	-11.9 (1.8)	-15.2 (1.9)	-11.8 (1.6)
	RANDOM	0.4 (2.4)	-0.9 (1.7)	-1.6 (1.9)	2.5 (2.4)

**10**					
	BM 1	-70.6 (4.1)	-31.9 (2.6)	-30.6 (2.7)	-37.2 (2.4)
	BM 5	-67.5 (4.7)	-31.0 (2.5)	-31.9 (1.9)	-33.8 (2.4)
	PLSR 1	-57.9 (5.2)	-39.5 (3.2)	-39.2 (2.4)	-40.1 (2.3)
	PLSR 5	-56.0 (7.3)	-37.9 (3.0)	-33.8 (2.9)	-36.9 (2.4)
	GBLUP 1	-56.8 (4.5)	-19.2 (4.2)	-20.0 (2.9)	-26.4 (2.6)
	GBLUP 5	-52.0 (5.2)	-18.8 (3.0)	-21.1 (3.1)	-25.8 (2.1)
	BLUP	-18.7 (6.3)	-13.0 (3.7)	-9.5 (3.8)	-13.5 (3.5)
	RANDOM	-7.4 (3.9)	2.1 (2.7)	-5.4 (2.9)	0.2 (2.7)

Cumulative change (standard deviation) in genetic variance, after one and ten generations of selection (as a percentage of the genetic variance in the reference population) in genetic architectures with a low number of QTL of unequal variance (column 3), a high number of QTL of unequal variance (column 4), a low number of QTL of equal variance (column 5) and a high number of QTL of equal variance (column 6); selection was on breeding values estimated with a Bayesian method (BM), partial least square regression (PLSR), genomic BLUP (GBLUP) or pedigree BLUP (BLUP), or selection was at random (RANDOM); numbers 1 and 5 behind estimation methods indicate the number of generations used in the training population

The final percentage of genetic variance remaining in generation 10 ranged from 29.4% with BM in the low-unequal genetic architecture to 90.5% with pedigree BLUP in the low-equal genetic architecture. Comparing between the genomic selection approaches, GBLUP was best, it retained the highest genetic variance (43.2% to 81.2%), PLSR the worst (42.1% to 66.2%) and BM (29.4% to 69.4%) roughly in the middle between GBLUP and PLSR, with the exception of the low-unequal architecture for which the lowest genetic variance was retained by BM.

In summary, GBLUP could retain the highest genetic variance while PLSR retained the lowest genetic variance, except in the low-unequal genetic architecture where BM retained up to 15% less genetic variance than GBLUP (Table 6). The deep reference population resulted in a smaller reduction in genetic variance after one generation of selection than the shallow reference population, but after ten generations, the differences in genetic variance were very small (Table 6).

## Discussion

In this study, response to selection was determined over ten generations with different selection approaches that combined one of the following estimation methods BM, BLUP, GBLUP or PLSR with a deep or shallow reference population structure. It has been found that accuracies of MEBV reduce with increasing distance between reference and selection candidates [7,17] and that selection increases the effect of distance on accuracy and hence, on response to selection [5,11]. The different selection approaches were compared under four different genetic architectures to investigate the effects of evaluation methods and reference population on accuracy of MEBV and selection response. The results of this study can help to choose MEBV methods for distinct scenarios.

### Breeding value estimation methods

Genetic architecture affects the comparison of methods to estimate genomic breeding values. In the frequently simulated low-unequal architecture, which has a few QTL, there is a clear benefit for the BM method. In the low-unequal scenario, BM appears to be able to identify markers in LD with QTL, giving this method an advantage in early generations and in long-term response. The increase in $\bar{\text{G}}$ with the PLSR method was comparable to results obtained with GBLUP in the low-unequal scenario. This result is different from the pattern observed in [29], where PLSR showed considerably lower accuracy than BM and GBLUP in a simulated dataset that was very similar to the low-unequal architecture used here. A reason for this difference could lie in the implementation of PLSR. The results in [29] were obtained by a two-step procedure where variable selection preceded model fitting, which is suboptimal to the simultaneous selection and fitting of the model that was applied to obtain the results presented here. When the number of QTL increases, as for the three genetic architectures other than low-unequal, the conclusions change. The three genomic methods performed differently in terms of genetic improvement, with GBLUP performing best in generation 1 and PLSR or GBLUP performing best in generation 10 for approaches that used a shallow or deep reference population, respectively (Table 3). GBLUP had a clear advantage in generation 10,especially in comparison to PLSR and BM, with a smaller increase in inbreeding and smaller reduction of genetic variance. The GBLUP method combined a good response in $\bar{\text{G}}$ with a smaller increase in $\bar{\text{F}}$.

Although the priors were set to the true values for genetic variance and number of QTL, which would be difficult in practice, BM resulted in somewhat smaller increases in $\bar{\text{G}}$ compared to the other genomic evaluation methods for all architectures except the low-unequal one where BM resulted in an intermediate increase in G. Low-unequal is an architecture that fits the approach of the BM model well, since having fewer QTL improves the power to select the correct SNP into the model [16,20].

Selection is an important factor when comparing methods to estimate genomic breeding values, especially for traits with a low-unequal architecture. In populations under selection, the pattern of decrease in accuracy was not very different between estimation methods. However, without selection in the RANDOM scenarios, BM performed better to keep high accuracies up to ten generations past the reference population. It is important to realize that this advantage disappears when one is actually selecting on the MEBV. Genomic selection approaches are expected to incur less inbreeding than pedigree BLUP selection [11,30]. When the estimation methods BM, PLSR and GBLUP became inaccurate in later generations, they caused much smaller increases in inbreeding compared to the pedigree BLUP method. The lower inbreeding from genomic estimation compared to pedigree BLUP agreed with earlier results that indicated that genomic estimation methods can track mendelian sampling within families [30] and that pedigree BLUP tends to select family members [31].

Accuracies of pedigree BLUP breeding values in generation 0, and hence the response to selection on pedigree BLUP in generation 1 were at the same level as accuracies and response for genomic selection methods. The pedigree BLUP accuracy in generation 1, of approximately 0.60, was as expected with a heritability of 0.25 and phenotypes on the selection candidates and several of its sibs. The accuracies for genomic evaluation methods depend, among other factors, on the size of the reference population. The reference population size was chosen to yield an intermediate accuracy to allow for differences in accuracies from estimation method and/or reference population structure to become evident. Accuracies that are obtained as an output of the genetic evaluation model, i.e. obtained from the mixed model equations in pedigree BLUP, can be biased if pre-selection occurs on for instance MEBV [32]. Similarly, the estimated genetic progress can be affected by bias in the accuracies of MEBV when they are obtained from the evaluation model. These biases were not found in our simulation results because accuracies were obtained from correlations of MEBV with the true breeding values and selection response was calculated as the increase in average true breeding values.

### Importance of reference population structure

Differences between reference populations with a deep or shallow structure were most apparent in the first generations of selection. Methods to estimate MEBV used not only the LD in the population but also any family structure within the reference population that was detectable by markers. When predicting MEBV in generation 1 with data from the shallow reference population, a considerable contribution to the accuracy of those MEBV will originate from family structure detected by markers [21]. Especially in a small breeding population, individuals may need to be included from multiple generations to make up a sizeable reference population. The deep reference population that covered multiple generations increased the average genetic distance of candidates with reference individuals and reduced the accuracy of the MEBV and resulting selection response in generation 1. In later generations, the advantage of the shallow reference population decreased and accuracies and levels of response became similar to those obtained with a deep reference population. In these later generations, the markers lost their ability to explain family structure, which appeared to benefit the shallow reference structure more. In later generations the deep reference structure probably benefited from having less focus on capturing family structure and better use of LD information but it was concluded that the impact of reference population structure on long-term response was small. Only in a few scenarios did we see the expected pattern where cumulative genetic gain from a deep reference structure overtakes the accumulated gain from the shallow reference structure. Early gains made by the shallow reference structure are difficult to overcome by the greater gains made in later generations with the deep reference structure. One reason may be that accuracy, and also genetic variance, declined over time, which made early gains even more important.

In contrast to the small impact of reference population structure found in our results, Muir [11] showed a large impact of reference population structure on accuracy of MEBV after one to eight generations of random selection. The result of Muir [11] was obtained in a simulated population in two-locus Hardy Weinberg equilibrium, which meant absence of LD between markers and between markers and QTL. A deep reference population, named TG4, made up of generations 1 to 4, was compared to a shallow reference population, named TG2, made up of generations 1 and 2. TG2 resulted in an accuracy that was about 15% lower compared to TG4 in the sixth generation after training. We expect that in these results, the more persistent accuracy from the deep reference population was due to the fact that TG4 had two more generations to build up LD after starting the population in linkage equilibrium. In addition, the effect of building up more LD in the TG4 compared to the TG2 scenarios was strenghtened by the smaller effective population size in TG4 (*N*_*e *_= 64) compared to TG2 (*N*_*e *_= 128). In our simulations, we kept *N*_*e *_equal and the same level of historic LD was present in the deep and shallow reference population structures.

### Selection strategy

In this study, we used information from a reference population with ten generations of selection to evaluate the long-term impact of reference population structure and the persistency of methods. Many other choices for genotyping and phenotyping strategies could have been made and selecting on the same marker effects for ten generations is not a practical application, given the low accuracies that were obtained after ten generations under all genetic architectures. One exception might be the low-unequal architecture, where genomic selection resulted in a reduction of up to 71% of genetic variance and re-training the model would not have much value. In all other scenario's, retraining the models after a number of generations is expected to considerably improve response in later generations, as was shown by Sonesson and Meuwissen [17]. Selection without retraining can still be of practical value. Traits that are difficult or expensive to measure can warrant the use of the same reference population for several generations. To address our main questions, the impact of estimation methods and reference population structure on long-term selection, we chose to simulate genomic selection scenarios without retraining. Retraining, or adding more generations with phenotypes would have obscured the assessment of the persistency of methods (i.e. the ability of a method to assign genetic variance to markers in close LD with the QTL) and would have reduced the contrast between the deep and shallow reference population by making both populations "deeper" each generation. It should be realized that without retraining, our results do not show the maximum potential of genetic progress from genomic selection but that was not the aim of this study.

### Inbreeding

Accumulation of inbreeding was calculated based on pedigree relationships. The pedigree measure of inbreeding is supposed to capture genome-wide increase in homozygosity but this may not be the most relevant measure if genetic variance is due to a few QTL, and selection changes allele frequencies at these specific genome positions. In this case, average homozygosity may increase only a little although the favorable QTL are (nearly) fixed. In this situation a direct measure of genetic variance may be more valuable to describe the opportunities that remain for response to selection. For traits that are not under selection, pedigree-estimated inbreeding will still be a reasonable measure, assuming that loci that affect fitness are located away from the QTL with allele frequencies rapidly changed by genomic selection.

### Accuracy

A number of studies have described the accuracy of MEBV for individuals that are up to six [4,7,33], nine [11,17], ten [21], or 19 [5] generations away from the reference population. Of these studies, only Muir [11], Sonesson and Meuwissen [17] and Jannink [5] applied selection based on the MEBV, while random selection was applied in the other studies. In the study of Muir [11], accuracy of MEBV decreased quickly when the number of generations between the reference and the evaluation population increased, because of the very small number of QTL that were simulated, comparable to our low-unequal genetic architecture. Therefore the resulting decrease in accuracy of the MEBV was largely due to the reduction in genetic variance. Any change in LD patterns may have played a minor role. In actual breeding programs, the reduction of genetic variance has been relatively small [34] and therefore changes in LD, due to drift and selection, are expected to play a much bigger role in reducing accuracy of MEBV in breeding programs that apply GS. The study by Sonesson and Meuwissen [17] showed a pattern of the decrease in accuracy and genetic response from their FIRST-GEN scenario, which is comparable to our results in the low-unequal scenario with BM. Their FIRST-GEN scenario was similar to our approach because it did not retrain the model. Their simulated genetic architecture was similar to our low-unequal architecture because QTL effects were sampled from a Γ(0.4,1.66) distribution which has a high density at low values. The study by Jannink [5] applied genomic selection to an inbred crop, and investigated the use of genomic breeding values prior to phenotyping. An increase in early selection gains was shown, especially when additional weight was placed on favorable alleles with low frequencies. The loss of favorable alleles was not evaluated in our study. In future research, we will extend the comparison of estimation methods and reference population structures for their effect on genomic parameters such as LD and allele frequencies of QTL. The differences seen in reductions of genetic variance for the different estimation methods indicate that these genomic parameters of LD and allele frequencies of QTL may be affected differently by different methods.

## Conclusions

Under selection, applying GBLUP leads to lower inbreeding and a smaller reduction of genetic variance especially in comparison to PLSR but also to BM, while a similar genetic improvement is achieved with these estimation methods for traits that have a moderate to large number of QTL. With a small number of large QTL, BM and PLSR were expected to result in greater response over ten generations of selection but differences were small and most progress was made by one of the scenarios that applied GBLUP. Without selection and with a small number of large QTL, accuracies of MEBV from BM remained high for 10 generations past the reference population and were always higher than accuracies from the other methods. When selection on MEBV was applied however, no important differences were seen among the methods. Response to selection on MEBV for traits with a small number of large QTL, a common simulation scenario in recent literature, was limited in the long-term by a rapid reduction of accuracy over time, which was caused by a strong reduction in genetic variance. When the trait was affected by more QTL, reduction of genetic variance was limited and the decline in accuracy was smaller. The structure of the reference population had a limited effect on long-term accuracy and genetic gain. Based on these results, use of a reference population made up of individuals that are most closely related to the selection candidates is recommended. This approach gave early benefits but in later generations, without updating marker effects, the estimation of marker effects based on less related reference individuals did not pay off.

## Competing interests

The authors declare that they have no competing interests.

## Authors' contributions

All authors were involved in the design of the study. AC and JWMB contributed equally to the work, programmed the simulations and wrote the manuscript. All authors read and approved the manuscript.
